# Chloroplast protein complexes identified by TurboID in *Chlamydomonas reinhardtii*

**DOI:** 10.1093/plphys/kiad368

**Published:** 2023-06-28

**Authors:** Jiawen Chen

**Affiliations:** Assistant Features Editor, Plant Physiology, American Society of Plant Biologists, Rockville, MD, USA; John Innes Centre, Norwich Research Park, Norwich, NR4 7UH, UK

Chloroplasts are the site of photosynthesis, providing autotrophic carbon fixation. Chloroplast proteome homeostasis is important for chloroplast development and requires molecular chaperones. Chaperones facilitate protein folding and prevent unfolded proteins from forming aggregates. They are important in stress responses, and many of them are called heat shock proteins (HSP) because of their roles in heat stress responses. There are 4 major families of chaperones: chaperonin60 (Cpn60), HSP70, HSP90, and HSP100. Chaperones perform their functions using ATP as the energy source (Trösch et al. 2015). HSP70 is highly conserved across species, and the chloroplast HSP70 is regulated by the interacting nucleotide exchange factor CGE1 (chloroplast GrpE homolog 1) and a J-domain co-chaperone (CDJ1-6). CDJ proteins stimulate the HSP70 ATPase activity and bring specific substrates for proper folding (Trösch et al. 2015).

One of the known substrates of the chloroplast HSP70 system is VESICLE-INDUCING PROTEIN IN PLASTIDS 1 (VIPP1). In *Chlamydomonas reinhardtii*, chloroplast HSP70 forms a HSP70B/CGE1/CDJ2 complex (Liu et al. 2007). VIPP1 is involved in thylakoid membrane biogenesis, synthesis of membrane protein complexes, and protection of chloroplast membranes from stresses such as high light and heat. *cr*VIPP1 assembles into large homo-oligomers that form rods around thylakoid membrane tubules. Some short rods have been observed between the chloroplast inner envelope and thylakoids, and they are hypothesized to function in lipid transfer (Gupta et al. 2021). *cr*VIPP1 assembly is dependent on the HSP70B/CGE1/CDJ2 chaperone system (Liu et al. 2007). *cr*VIPP1 also interacts with the VIPP1 paralog VIPP2 under stress, and *cr*VIPP2 binds more strongly to chloroplast membranes than *cr*VIPP1 under H_2_O_2_ stress (Theis et al. 2020). VIPP1/2 are proposed to alleviate membrane curvature elastic stress.

To understand how VIPP1/2 regulate chloroplast membrane and envelope structures, we need to identify their interaction partners in the native context and learn what changes occur under environmental stress. Proximity labeling (PL) is a powerful technique for detecting protein–protein interactions in a native context. A main advantage of PL over traditional methods such as affinity purification mass spectrometry is that it can capture transient interactions. In PL, a bait protein is fused to a biotin ligase enzyme that activates biotin and covalently links biotin to proteins in proximity to the bait. The labeled proteins can be purified using streptavidin beads and further analyzed by immunoblotting or mass spectrometry (MS). PL is commonly performed using 1 of 2 enzymes: BirA and APEX. An optimized version of PL with BirA was named BioID (proximity-dependent biotin identification), and TurboID was developed as a higher-efficiency version of BioID (Branon et al. 2018).

PL has been used successfully in land plants (Mair et al. 2019), where protein–protein interactions were analyzed in both whole cell and nuclear proteomes. However, chloroplast-specific PL had not yet been performed. In this issue of *Plant Physiology*, Kreis et al. (2023) successfully applied PL in *C. reinhardtii* chloroplasts, focusing on the transient interactions of proteins with HSP70B. They showed the effectiveness of TurboID in capturing HSP70B using both CGE1 and VIPP1 as bait under ambient and stress conditions. In addition to the known HSP70 interactors, the authors also discovered new partners of the HSP70B/CGE1/CDJ2 system that were not previously captured by other methods, such as CGE2 as a novel co-chaperone.

The authors tested several different PL enzymes, including APEX2, BioID, and TurboID. They first used CGE1 bait constructs to test whether biotin labeling could be captured. APEX2 did not result in any biotin labeling, BioID resulted in weak labeling, and TurboID had the most efficient self-labeling. Therefore, the authors conducted further experiments with TurboID constructs of CGE1, VIPP1, VIPP2, and mCherry control to test for known interactions such as CGE1 or VIPP1 with HSP70B. Using these constructs, in vivo self-biotinylation could be detected without exogenous biotin application, but with added biotin this labeling drastically increased; addition of 1 mM biotin for 1–6 h was the optimal condition. Of note is that native biotinylation levels were proportionally decreased to TurboID expression levels because of competition for endogenous biotin, but this does not seem to affect cellular fitness under high light and temperature stress.

To capture the CGE1 and VIPP1/2 PL proteomes, the authors performed TurboID and detected biotin-labeled proteins in proximity to the bait by immunoblotting for specific candidates using streptavidin-HRP and target-specific antibodies and by performing LC-MS/MS analyses on the streptavidin eluates to detect any other associated proteins. They also compared the proxiomes under ambient and stress conditions, using heat stress (40 °C) for the CGE1 proxiome and H_2_O_2_ stress for the VIPP1/2 proxiomes.

Both known and new protein interactions were captured in the CGE1 and VIPP1/2 proxiomes (Fig.). For the CGE1 proxiome, no exogenous biotin was applied, and known members of the *C. reinhardtii* HSP70 system were detected: HSP70B and the co-chaperone CDJ1. CGE2 was captured as a novel co-chaperone, and the authors hypothesized that CGE1 and CGE2 could form heterodimers. These interactions were detected in both ambient and heat-stressed conditions. The mitochondrial HSP70C was detected in the CGE1 proxiome under non-stress conditions, suggesting it may be dually localized.

**Figure.**
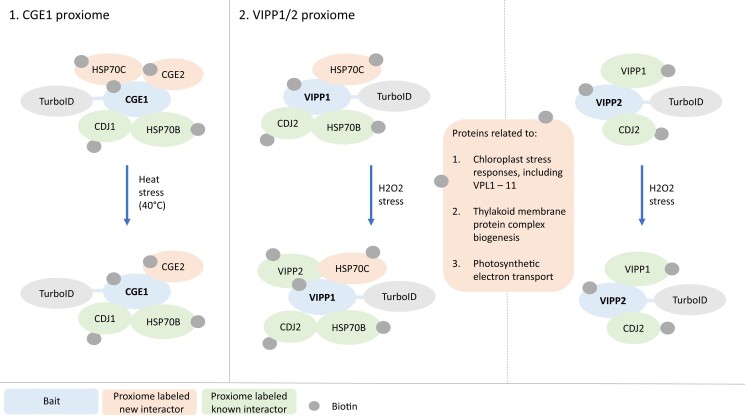
Turbo-ID in *C. reinhardtii* chloroplasts identifies known and new protein interactors in the CGE1 and VIPP1/VIPP2 proxiomes in ambient and stress conditions. The main protein interactions highlighted in this study are summarized in this figure. These interactions were identified by LC-MS/MS analysis and some also by immunoblotting. There are additional proteins detected in the proxiomes by LC-MS/MS that are not included in this figure, for which not much is known. Using TurboID-CGE1 as bait, the known interactors HSP70B and CDJ1 of the HSP70B chaperone system were detected, confirming the robustness of this TurboID system in *C. reinhardtii* chloroplasts. This technique also captured the previously unidentified co-chaperone CGE2 in the CGE1 proxiome. The VIPP1/VIPP2 proxiomes captured 39 interactions. The interaction of VIPP1 with HSP70B and CDJ2 was confirmed in the VIPP1-TurboID proxiome, and VIPP1–VIPP2 interaction was also detected under H_2_O_2_ stress. VIPP2 is expressed at lower levels than VIPP1, and VIPP2 interaction with HSP70B was not detected here. In both the CGE1 and VIPP1 proxiome, the mitochondrial HSP70C was detected, suggesting dual localization in mitochondria and chloroplasts. In addition to the specifically highlighted interactions above, VIPP1/2 proxiomes included proteins related to chloroplast stress, thylakoid membrane complex biogenesis, and photosynthetic electron transport.

For the VIPP1/2 proxiomes, the authors used 3 different biotin labeling protocols: without exogenous biotin, in vitro biotin labeling with 500 µM biotin added to crude membrane extracts, and in vivo labeling with 1 mM biotin. For the latter 2 conditions, parallel experiments under H_2_O_2_ stress were also performed. In summary, 39 proteins were enriched in the VIPP1/2 proxiomes. The authors confirmed the VIPP1–VIPP2 interaction in both proxiomes, where VIPP2 was only captured with the VIPP1 bait under oxidative stress. They also confirmed the interaction of both VIPP1 and VIPP2 with CDJ2, but HSP70B was enriched with VIPP1-TurboID only. Of the 39 total enriched proteins, 17 of the corresponding genes were upregulated by stress in previous studies, with 11 of these with no clear functional annotation. These 11 proteins were named VIPP PROXIMITY LABELING (VPL) 1–11. The other 22 proteins were mainly involved in the biogenesis of thylakoid membrane protein complexes and photosynthetic electron flow, and a few proteins had individual functions. Two of the VIPP1 interactions were also confirmed in reciprocal TurboID experiments, VPL2 and PGRL1, one of the photosynthetic electron flow proteins.

This study presents a new use of TurboID in the *C. reinhardtii* chloroplast and demonstrated a useful tool for investigating HSP70B-associated proteins, including its substrates VIPP1/2. This expands on the plant subcellular compartment repertoire in which TurboID can be effectively used. Two parallel studies on chloroplast PL were also conducted on the *C. reinhardtii* pyrenoid proxiome (Lau et al. 2023) and in Arabidopsis developing chloroplasts (Wurzinger et al. 2022). Together, these 3 studies demonstrate the great potential of using PL in plant organelles.
